# Hydrogen Bonding Penalty upon Ligand Binding

**DOI:** 10.1371/journal.pone.0019923

**Published:** 2011-06-17

**Authors:** Hongtao Zhao, Danzhi Huang

**Affiliations:** Department of Biochemistry, University of Zurich, Zurich, Switzerland; Anne Arundel Community College, United States of America

## Abstract

Ligand binding involves breakage of hydrogen bonds with water molecules and formation of new hydrogen bonds between protein and ligand. In this work, the change of hydrogen bonding energy in the binding process, namely hydrogen bonding penalty, is evaluated with a new method. The hydrogen bonding penalty can not only be used to filter unrealistic poses in docking, but also improve the accuracy of binding energy calculation. A new model integrated with hydrogen bonding penalty for free energy calculation gives a root mean square error of 0.7 kcal/mol on 74 inhibitors in the training set and of 1.1 kcal/mol on 64 inhibitors in the test set. Moreover, an application of hydrogen bonding penalty into a high throughput docking campaign for EphB4 inhibitors is presented, and remarkably, three novel scaffolds are discovered out of seven tested. The binding affinity and ligand efficiency of the most potent compound is about 300 nM and 0.35 kcal/mol per non-hydrogen atom, respectively.

## Introduction

Hydrogen bonding is an exchange reaction whereby the hydrogen bond donors and acceptors of the free protein and ligand break their hydrogen bonds with water and form new ones in the protein-ligand complex [1], [2], [3]. About thirty years ago, Wilkinson and coworkers found mutation of Cys-35 in Tyrosyl-tRNA synthetase to Ser-35 causes poorer ATP binding and catalysis although the hydroxyl group of serine forms far stronger hydrogen bonds than does the thiol group of cysteine [1]. Analysis of the hydrogen bonding geometry revealed that a hydrogen bond of Ser-35 is at least 0.5 Å longer than the optimum. Accordingly, Ser-35 would have to lose a good hydrogen bond with a bound water molecule to form this weak hydrogen bond with ATP in the enzyme-substrate complex, and thus the mutant shows poorer binding and catalysis. Therefore, enthalpic loss in hydrogen bonding could take place upon ligand binding if not compensated by formation of good hydrogen bonds between the protein and ligand.

Virtual screening has emerged as an efficient tool in drug discovery from lead identification to optimization and beyond [4], [5]. However, scoring functions that model the solvent environment as a continuum [6], [7] are still grossly inaccurate [8]. The role of individual waters can be critical in predication of binding affinities, and continuum models often provide poor results in treating bound waters in a confined cavity [9]. Glide docks explicit waters into the binding site and measures the exposure of polar/charged groups to the explicit waters. When a polar/charged ligand or protein group is judged to be inadequately solvated, a desolvation penalty is assessed [9], [10]. By contrast, most other scoring functions [11] do not properly take into account the enthalpic loss of hydrogen bonding upon ligand binding. Incorporation of bound water molecules into molecular docking was suggested for improvement of accuracy [12]. On the other hand, in high-throughput molecular docking campaigns a significant part of binding poses are rather unrealistic, e.g. burial of polar atoms in hydrophobic sites, and thus discarding them at an early stage is desirable. Filters such as van der Waals efficiency based on arbitrary cutoff are often used to remove poses that unlikely bind [13]. However, it seems lack of a reliable and efficient filter with transferable cutoff among different proteins.

Protein kinases play an important role in cell-signaling pathways regulating a variety of cellular functions. Dysregulation of kinase activity has been implicated in pathological conditions ranging from neuronal disorders to cellular transformation in leukemia [14]. The tyrosine kinase erythropoietin producing human hepatocellular carcinoma receptor B4 (EphB4) is involved in cancer related angiogenesis [15]. So far, two high-throughput virtual screening campaigns have been reported, with two scaffolds identified in the low micromolar range [13], [16]. Highly potent EphB4 inhibitors have been developed via chemical synthesis [17], [18], [19]. The marketed drug dasatinib, with Abl1 and Src as primary targets, also shows a very high affinity to Eph kinases [20].

Here, we report a new approach to calculate hydrogen bonding penalty (HBP) associated with ligand binding. HBP is further integrated into a binding energy calculation, and the fitted parameter of 1.7 kcal/mol is consistent with the estimate of contribution by formation of one neutral hydrogen bond ranging from 0.5 to 1.5 kcal/mol [21]. Moreover, statistics of HBP in kinase crystal structures and an application in a high-throughput docking campaign is presented.

## Methods

Binding of a ligand to a protein involves the breakage of hydrogen bonds with water molecules and formation of new hydrogen bonds between the protein and ligand, which can be described by the following equation [21] by using one pair of donor (D) and acceptor (A):
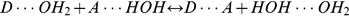
(1)Based on hydrogen bonding being an exchange reaction [1], [21], [22], its energy can be described using normalized weights:
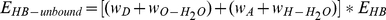
(2)

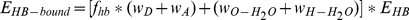
(3)wherein, *w_D_* and *w*_A_ is the hydrogen bonding weight of a donor or acceptor, respectively, *f*_hb_ stands for the fraction of hydrogen bonding relative to that of an optimum geometry, and *E*_HB_ is unit hydrogen bonding energy. Hydrogen bonds with water are assumed to be in the optimum geometry. HBP (*p*_HB_) associates with ligand binding is then described as
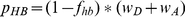
(4)


### Probing hydrogen bonding status

Oxygen and nitrogen atoms in double or triple bonds are regarded as hydrogen bond acceptors, and hydrogen atoms bonded to oxygen, nitrogen or sulfur atoms are regarded as hydrogen bond donors. The existence of C—H…O hydrogen bonds has been confirmed by neutron diffraction data on organic compounds [23]. Analysis of 100 kinase crystal structures complexed with small molecule inhibitors at a resolution of at least 2.5 Å gives 64 short C—H…O interactions, showing typical hydrogen bonding features (Figure S1).

**Figure 1:**
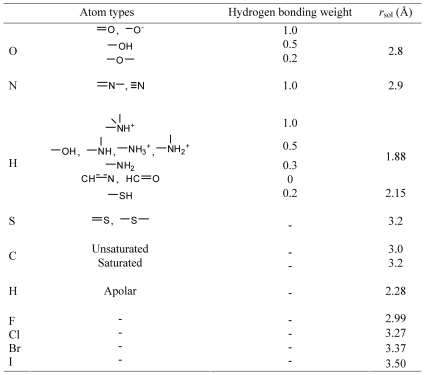
Hydrogen bonding weights and solvation radii of different atom types.

Each hydrogen bond donor or acceptor at the binding interface is firstly checked whether it forms hydrogen bond with water molecules. For this purpose, an optimum solvation radius (*r*_sol_) is defined for each donor/acceptor and if a water molecule can be placed within 0.15 Å of the *r*_sol_ no penalty is applied. Here, 2.8 and 2.9 Å are used as *r*_sol_ for any oxygen and nitrogen, respectively, which were derived from an analysis of 397 crystal structures with X-ray resolutions below 1.0 Å (Figure S2). The *r*_sol_ of polar hydrogen is 1.9 Å (except 2.15 Å for H bonded to sulfur), which is the difference between the *r*_sol_ of nitrogen and the bond length [24] of N—H. The *r*_sol_ of other atom types are listed in Figure 1 and the values are mainly adapted based on the van der Waals radii of Bondi [25]. Details of probing hydrogen bonds with water were described in File S1. In case of not forming hydrogen bonds with water, the possibility of forming hydrogen bonds between the protein and ligand (including intra-molecular hydrogen bonds) is further checked and penalty (*p*_HB_) is then calculated.

### Fraction of hydrogen bonding

Similar to the strategy of evaluating hydrogen bonding energy in LUDI [26], the following equations are used to calculate the fraction of hydrogen bonding (*f*_hb_) to that of an optimum geometry.
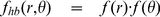
(5)

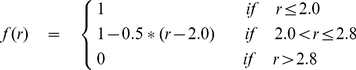
(6)

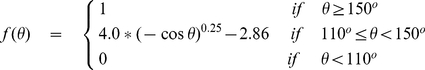
(7)wherein, *r* is the distance between the hydrogen atom and the acceptor and *θ* is the angle centered at hydrogen among donor, hydrogen and acceptor. The equation to calculate *f(r)* and *f(θ)* as well as the upper and lower limit in *r* and *θ* are derived from the calculation using density functional theory [27]. In case of one hydrogen atom is shared by two acceptors or one acceptor interacting with two donors, the *f*_hb_ for the corresponding donor/acceptor is additive but with 1 as the upper limit.

### Hydrogen bonding penalty

The HBP at the protein-ligand interface is summarized over each donor/acceptor as
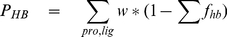
(8)However, no penalty is applied for protein atoms which are not water accessible before ligand binding or participate in intra-molecular hydrogen bonds. Initial guess of hydrogen bonding weights (*w*) is based on chemical intuition by considering atomic partial charge and water solubility of a few small molecules (Table S1). Empirical weights as proof-of-principle are then optimized with a trial-and-error procedure according to the fitted parameter in the binding free energy calibration.

### Evaluation of binding free energy

The equation used for fitting the calculated energies to the experimental free energies of binding (Δ*G* = *RT*ln(*K_d_*)) is a three-parameter model
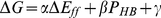
(9)where, Δ*E*_ff_ is the interaction energy between the ligand and the protein calculated by the CHARMm force filed [28] and *P*_HB_ stands for HBP. Three parameters α, β, and γ are generated with fitting. Δ*E*_ff_ is calculated by the following equation:
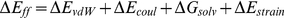
(10)where, Δ*E*_vdW_ is the intermolecular van der Waals energy, Δ*E*_coul_ is the intermolecular Coulombic energy in vacuo, Δ*E*_strain_ is the strain energy of ligand upon binding, and Δ*G*_sol_ is the change in solvation energy of ligand and protein upon binding.

The van der Waals and Coulombic interaction energy are calculated by subtracting the values of the isolated components from the energy of the complex with CHARMM [29] and the CHARMm22 force filed [28]. The van der Waals energy is calculated using the default nonbonding cutoff of 14 Å. Coulombic energy is calculated using infinite cutoff and a dielectric constant of 2.0. The electrostatic solvation energy was calculated by the finite-difference Poisson approach (FDP) [30] using PBEQ module [31] in CHARMM and a focusing procedure with a final grid spacing of 0.25 Å. The size of the initial grid is determined by considering a layer of at least 12.5 Å around the solute. The dielectric discontinuity surface was delimited by the van der Waals surface. The ionic strength is set to zero and the temperature to 300 K. Two finite-difference Poisson calculations are performed for each of the three systems (protein, ligand, and protein/ligand complex). The exterior dielectric constant was set to 78.5 and 2.0 for the first and second calculation, respectively, while the solute dielectric constant is 2.0 to take polar fluctuations into account. The solvation energy is the difference between the two calculations. The strain energy of the ligand is the energy difference between the bound and global minimum. Here, the global minimum is the one showing the lowest *E*_vdW_+*E*_coul_+*E*_b*onded*_+*G*_sol_ among all the poses that have been minimized outside of the protein.

Twenty-three inhibitors [32] of CDK2 (1H0V), 24 inhibitors [18] (**8** to **32**, excluding **30**) of EphB4 (2VWX), and 27 uncharged inhibitors [33] of p38 alpha MAP kinase (3GC7) are used as the training set. Thirty type II inhibitors [34] of Braf (3II5), 14 charged inhibitors [33] of p38 alpha and another 20 p38 alpha inhibitors [35] (1YWR) are used as the test set. Protein structures were taken from the X-ray structure (PDB code indicated in the brackets) and prepared as described below. Some key physiochemical properties of inhibitors are summarized in Figure S3.

Version 4 of AutoDock [36] was used to generate the binding poses over the conformational search space using the Lamarckian genetic algorithm. The binding site was determined by 4.0 Å away from any atom of the ligand complexed in the respective protein structure. The number of energy evaluations was 2,750,000 and the number of poses was 50. Poses were further clustered using all atom RMSD cutoff of 0.3 Å to remove redundancy and in average 20 cluster representatives were kept. All other parameters were set as default. A few poses for each inhibitor were also generated by manual modification of the scaffold present in the respective crystal structure. All poses were further minimized by CHARMM in the respective proteins. The protein structure was kept rigid in all steps.

### Preparation of protein-ligand complexes

One hundred kinase crystal structures (including 15 different classes, File S2) complexed with small molecule inhibitors at a resolution of at least 2.5 Å were downloaded from Protein Data Bank for analysis of HBP. Hydrogen atoms were added according to the protonation states of chemical groups at pH 7. Partial charges were then assigned using MPEOE method [37], [38]. The added hydrogen atoms were minimized by the conjugate gradient algorithm to a RMS of the energy gradient of 0.01 kcal mol^−1^ Å^−1^. During minimization, the electrostatic energy term was screened by a distance-dependent dielectric of 4*r* to prevent artificial deviations due to vacuum effects, and the default nonbonding cutoff of 14 Å was used. Furthermore, the positions of all heavy atoms were fixed.

### Preparation of the compounds library for virtual screening

The compounds were selected from Zinc library [39]. Preparation included the assignment of CHARMm atom types, force field parameters [28], and partial charges [37], [38], and energy minimization with a distance dependent dielectric function using the program CHARMM [29].

### Enzymatic assay

In vitro kinase activity was measured using the Panvera Z'lyte Tyr2 kinase assay PV3191 (Invitrogen) according to the manufacturer's instructions. The reaction assay (10 µL) contained 7.5 ng of EphB4 kinase (Proqinase, Germany), 30 µM ATP, and 5% DMSO. The reaction was performed at room temperature for 1 h.

## Results and Discussion

### Statistics of hydrogen bonding penalty in kinase complexes

Small HBPs can be observed for the binding modes of inhibitors in the X-ray structures. One example is c-Kit tyrosine kinase with its apo and holo form in complex with Imatinib (PDB codes 1T45 and 1T46). In the apo conformation, donors/acceptors at the ATP binding site form hydrogen bonds with bound water molecules. While upon ligand binding, as shown in the holo conformation, some water molecules are displaced by Imatinib. HBP on the protein part is close to zero because new hydrogen bonds to the protein are formed to compensate for the replacement of the water molecules. However, one nitrogen atom of the Imatinib pyrimidine ring (N1 of Figure S4) becomes water inaccessible and does not form a new hydrogen bond, leading to a penalty of 1. By contrast, the other nitrogen atom (N2 of Figure S4) remains hydrogen bonding with a nearby bound water molecule and thus has no penalty.

**Figure 2:**
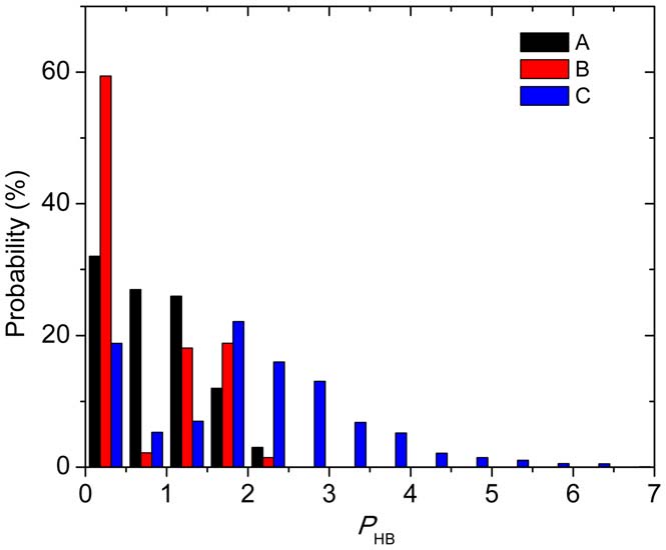
Distribution of hydrogen bonding penalties for: A) the binding modes in crystal structures of the 100 kinase complexes; B) poses with the most favorable calculated binding energies of the 138 molecules used in binding free energy calibration; C) all poses of the 138 molecules.

To check the distribution of HBP values in crystal structures, 100 kinase-ligand complexes are investigated. In this data set, all the small molecule inhibitors have molecular weights from 200 to 700 g/mol and number of donors or acceptors from 2 to 11 (File S2). The HBP has been calculated for each of them and the values are in general small, with 62% smaller than 1 and 36% and 2% in the range from 1 to 2 and 2.0 to 2.1, respectively (Figure 2 and File S2). It has also been observed that larger HBPs appear in some X-ray structures, e.g., the structures of PDB code 3KVX and 1JSV, and the large values actually originate from poor fitting of small molecules to the density, a common problem in crystallography [40] which can be manifested by clash of atoms.

Distribution of HBPs for docked poses of small-molecule inhibitors is also evaluated. Here, the 138 molecules used in the binding free energy calibration are docked into the corresponding protein binding sites with AutoDock. For each molecule, about 20 poses in average are generated. Then the HBPs and binding energies are calculated for all the poses. Firstly, the binding pose with the most favorable binding energy for each molecule (Figure S5) is selected and the distribution of HBPs is plotted. As can be observed from **B** of Figure 2, the distribution is similar to that of the 100 kinase complex structures (**A**). On the other hand, the distribution of all poses (**C**) spreads more widely with the largest HBP being 6.5. Compared with the HBPs in the crystal structures (**A**), 2 is a reasonable threshold, and about 50% of poses with unrealistic binding modes can be filtered out from further evaluations.

### Hydrogen bonding penalty improves the accuracy of binding energies calculation

Binding energies can be calculated using equation 9 with the parameters obtained by least-squares fitting on the training data set of the 74 CDK2, EphB4, and p38α inhibitors as following:
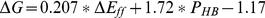
(11)


**Figure 3:**
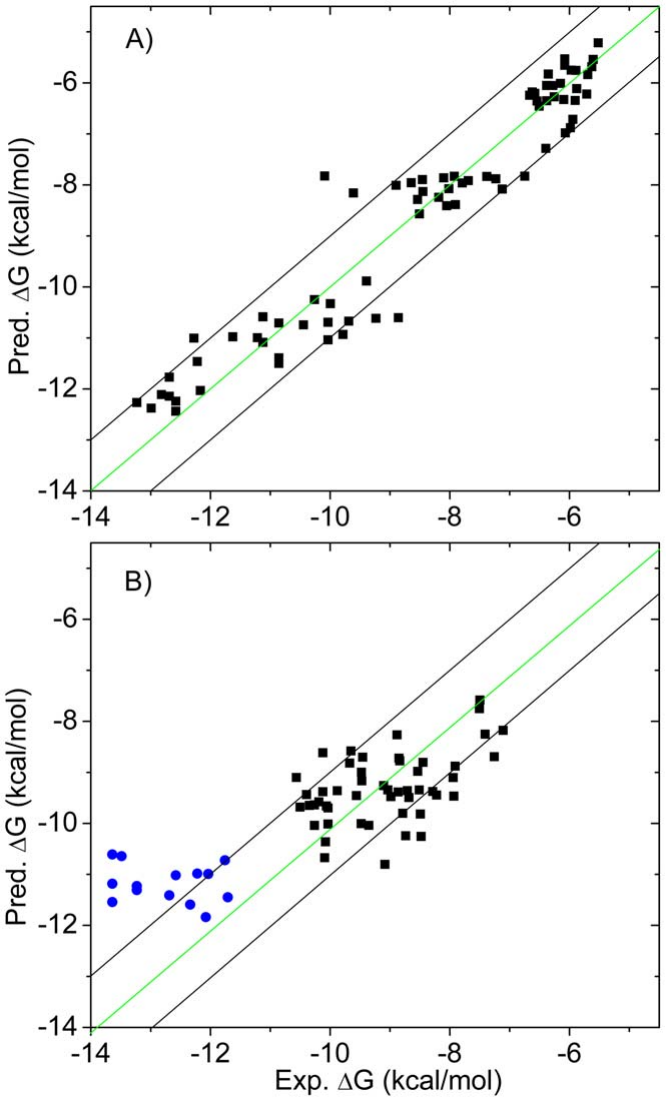
Comparison of the calculated versus experimental binding energies. A) Training set of 74 inhibitors. R^2^ = 0.92 and RMS error = 0.69 kcal/mol; B) Validation set of 64 inhibitors. RMS error = 1.12 kcal/mol. The blue dots indicated the 14 p38α inhibitors with one formal charge. The green diagonal line is the ideal line of perfect prediction. The black diagonals delimit the 1 kcal/mol error region.

The calculated binding energies show high correlation with the experimental values (R-square of 0.92) and a small RMS error of 0.69 kcal/mol (Figure 3A). Here, the parameter *β* corresponds to the unit hydrogen bonding energy. Notably, the fitted value 1.72 kcal/mol is in agreement with the experimental value, e.g., breakage of a neutral hydrogen bond resulting in loss of energy from 0.5 to 1.5 kcal/mol [21]. Moreover, a charged primary amine or carboxyl group has a hydrogen bonding weight of 1.5 or 2.0, which can lead to a maximal penalty of 2.58 or 3.44 kcal/mol upon loss of the hydrogen bond/salt bridge. This value also agrees well with the experimental data (up to 4 kcal/mol) [21]. Hydrogen bonding weights were further used to rank the strength of individual hydrogen bonds in DNA base pairs, exhibiting good compatibility with the previously reported results (File S3).

The fitted model has been validated on a test set including 14 charged p38α inhibitors and 30 type II Braf inhibitors, with an RMS error of 1.12 kcal/mol (Figure 3B). Moreover, validation with different kinases shows general transferability of this model (Table 1). Transferability can be also seen for aspartic protease, e.g., HIV-1 protease and β-secretase, although a shift of 2.0 kcal/mol can be observed for the latter. Previously, we reported a two-parameter LIECE model for kinase inhibitors [13], which is not transferable for type II kinase inhibitors, HIV-protease or β-secretase inhibitors. The binding affinities predicted by the two-parameter LIECE on the 24 type I EphB4 inhibitors show about −5.0 kcal/mol shift compared with the experimental values (Table S2). Clearly, the incorporation of HBP into the scoring function improves the general transferability besides the role of ligand reorganization energy [41].

**Table 1:** Further validation of the three-parameter model with kinases and aspartic protease.

Protein	PDB code	Δ*E*_ff_ (kcal/mol)	*P*_HB_	Δ*G*_pred_ (kcal/mol)	Δ*G*_exp_ (kcal/mol)
Abl	1OPJ	−64.80	1.24	−12.45	−10.81
Braf	1UWH	−57.61	1.27	−10.91	−10.45
JAK2	3E63	−30.18	0.00	−7.41	−7.91
Lck	2OFV	−59.13	0.53	−12.51	−13.23
JNK3	1PMV	−30.16	0.17	−7.12	−9.31
Ret	2X2L	−26.67	0.07	−6.58	−7.20
EGFR	1XKK	−66.60	2.30	−11.00	−10.91
CSrc	3G5D	−52.34	1.64	−9.19	−12.82
HIV-1 protease	1HIH	−65.71	1.49	−12.21	−11.01
	1HPX	−65.44	1.43	−12.26	−12.46
	1HXB	−61.24	0.95	−12.21	−13.49
	1HXW	−72.66	1.41	−13.78	−14.71
BACE-1	2QMF	−73.62	1.56	−13.72	−11.63
	2QP8	−68.76	0.47	−14.59	−11.05
	2XFI	−71.10	2.36	−11.83	−10.67

The derived model includes calculation of solvation energy by FDP which requires about 6 min on a single Intel 2.8 GHz CPU. Replacing the FDP approach with a distance-dependent dielectric model for solvation energy calculation gives similar accuracy for the neutral inhibitors at a much fast speed (10 seconds). However, distance-dependent dielectric model can only apply for non-charged compounds due to inaccurate treatment of the solvation effect, and also more false positives in a high-throughput virtual screening are observed. This comparison indicates that accurate calculation of solvation energies in prediction of binding affinities is necessary.

### Virtual screening for EphB4 inhibitors

In a recent high throughput docking study for EphB4 inhibitors, ZINC “leads-now” library of about 20 million compounds (Mw≤350 and cLogP≤3.5) was first tailored by a pharmacophore model to generate a focused library of 103,177 compounds. This pharmacophore model was specifically designed for EphB4 type I inhibitors, consisting of a bi-dentate hydrogen bonding pattern and a conjugate hydrophobic group to be located in the deep ATP back pocket as well as geometric constraints thereof (H. Zhao, unpublished results). To our best knowledge, all known type I EphB4 inhibitors [13], [16], [17], [18] can fulfill this model.

**Figure 4:**
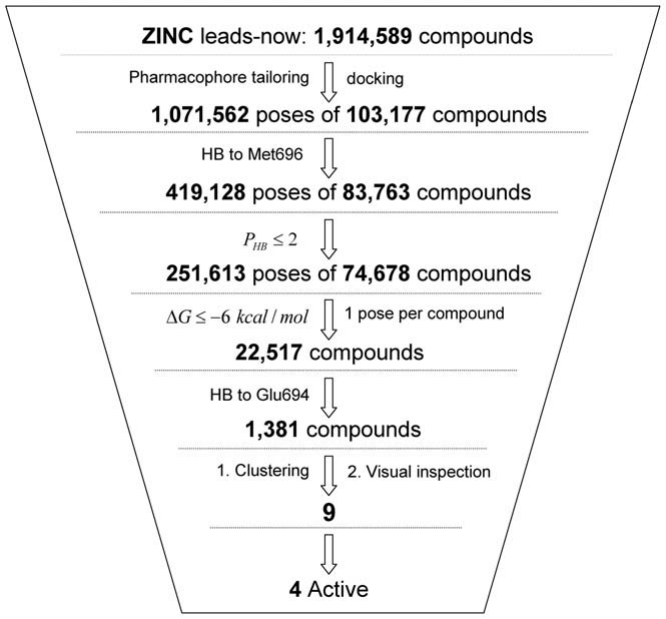
Schematic picture of the high throughput docking approach. HB stands for hydrogen bond. Met696 and Glu694 are the two key residues of the hinge loop (see also Figure 6).

The focused library was docked by AutoDock 4 and about 1 million poses were generated by clustering with a RMSD cutoff of 1.0 Å. The cluster representatives which do not form a hydrogen bond to NH of Met696 were further filtered out. The HBP (≤2) was then used to remove unrealistic poses (about 40%). The remaining poses were further ranked by the predicted binding energy, and the top about 30% compounds (22,517) with calculated binding energy smaller than −6 kcal/mol (∼50 µM) were kept. Among them, 1381 compounds forming a hydrogen bond to Glu694 were selected and can be classified into 80 structural scaffolds. Finally, 7 scaffolds (9 compounds) of them were purchased for experimental measurements based on visual inspection of the binding modes, commercial availability and structural novelty. The procedures used in the virtual screening are shown in Figure 4. Comparison of the performances between the proposed and AutoDock 4 scoring function is shown in Figure S6.

**Figure 5:**
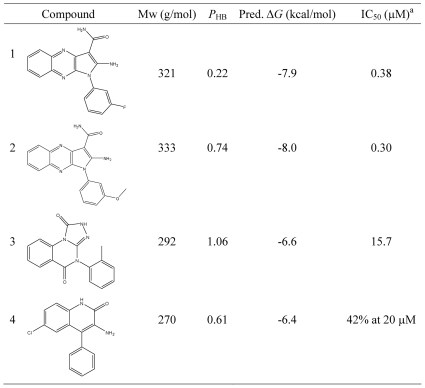
Identified EphB4 inhibitors by high throughput docking. ^a^ All IC_50_ values are means of two to four dose-response measurements.

**Figure 6:**
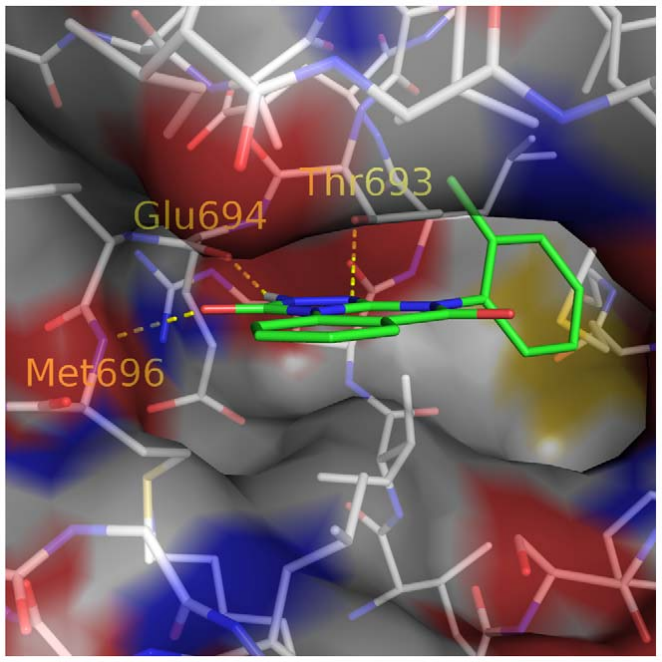
Binding mode of compound 3 (carbon atoms in green) predicted by docking. The intermolecular hydrogen bonds to the residues at the hinge loop (Glu694 and Met696) and the gatekeeper (Thr693) are shown by yellow dashed lines. The protein surface is colored based on atom types with carbon in white, oxygen in red, and nitrogen in blue. This figure was prepared using PyMOL (Delano Scientific, San Carlos, CA).

Notably, 4 of the 9 tested compounds show inhibitory activity at micro-molar to high nano-molar range, with the most active compound showing IC_50_ at 300 nM (Figure 5). Interestingly, the two compound also show a high ligand efficiency [42] of −0.35 kcal/mol per non-hydrogen atom. The predicted binding mode of compound **3** (Figure 6) is further confirmed by the preliminary X-ray crystallography (J. Dong, unpublished results).

### Conclusion

Hydrogen bonding in biological system is a complex phenomenon as water competes with ligand for the hydrogen bonding sites. Removal of a group that forms a hydrogen bond in unfavorable geometry actually improves binding [21]. In view of hydrogen bonding being an exchange reaction [1], [21], [22], a new approach is proposed to evaluate the HBP upon ligand binding. Analysis of the 100 crystal structures indicates the penalty in general is low, predominantly smaller than 2 for inhibitors. A high throughput docking case shows HBP can function as an efficient filter to remove poses that unlikely bind. Incorporation of HBP into binding free energy calculation can significantly improve the predictive accuracy and transferability. The fitted parameter of 1.72 kcal/mol means loss of a neutral hydrogen bond would result in a penalty of from 0.34 to 1.72 kcal/mol in binding energy, consistent with the experimental data from 0.5 to 1.5 kcal/mol [21]. Four inhibitors of three scaffolds were discovered out of nine tested, and the binding affinity and ligand efficiency of the most potent compound is about 300 nM and 0.35 kcal/mol per non-hydrogen atom, respectively.

## Supporting Information

Figure S1 **Scatter plot of C—H…O angles against H…O distances in short C—H…O interactions between ligands and proteins.**
(DOC)

Figure S2 **Distribution of distances between crystal water oxygen and oxygen or nitrogen atoms of proteins.**
(DOC)

Figure S3 **Distribution of some key properties of the inhibitors used in the training and test set.**
(DOC)

Figure S4 **2D plot of the binding mode of Imatinib.** Upon ligand binding, one nitrogen atom of the Imatinib pyrimidine ring (N1) becomes water inaccessible and does not form a new hydrogen bond, leading to a penalty of 1. By contrast, the other nitrogen atom (N2) remains hydrogen bonding with a nearby bound water molecule and thus has no penalty.
(DOC)

Figure S5 **Poses with the most favorable binding energy of inhibitors of CDK2 (A), EphB4 (B), p38 α (C), Braf (D) and another set of p38α inhibitors (E).** The molecules with bonds in red are the binding modes of the corresponding scaffolds in the crystal structures.
(DOC)

Figure S6 **Distribution of predicted binding affinities by Autodock4 (black) and the proposed scoring function (red) on 74,678 compounds passing the first two filters (HB to Met696 and** ***P*** **_HB_≤2 kcal/mol).** Bin size: 0.1 kcal/mol.
(DOC)

Table S1 **MPEOE partial charge and water solubility of model small molecules used to generate initial guess of hydrogen bonding weights.**
(DOC)

Table S2 **Two-parameter LIECE energy and hydrogen bonding penalty on the 24 EphB4 inhibitors.**
(DOC)

File S1 **Probing hydrogen bonds formed with implicit water.**
(DOC)

File S2 **Hydrogen bonding penalty of the 100 kinase complex structures.**
(DOC)

File S3 **Ranking the strength of individual hydrogen bonds in DNA base pairs.**
(DOC)
