# Supplementary material for: Hydrogen Bonding Penalty upon Ligand Binding
Source: PLoS One. 2011 Jun 17;6(6):e19923. doi: 10.1371/journal.pone.0019923 (PMC3117785; doi:10.1371/journal.pone.0019923)
Supplement: Figure S1 — Scatter plot of C—H…O angles against H…O distances in short C—H…O interactions between ligands and proteins. (DOC) [file pone.0019923.s001.doc]

**Figure S1.** Scatter plot of C―H∙∙∙O angles against H∙∙∙O distances in short C―H∙∙∙O interactions between ligands and proteins.
