# Supplementary material for: Hydrogen Bonding Penalty upon Ligand Binding
Source: PLoS One. 2011 Jun 17;6(6):e19923. doi: 10.1371/journal.pone.0019923 (PMC3117785; doi:10.1371/journal.pone.0019923)
Supplement: Figure S3 — Distribution of some key properties of the inhibitors used in the training and test set. (DOC) [file pone.0019923.s003.doc]

**Figure S3.** Distribution of some key properties of the inhibitors used in the training and test set.
