# Supplementary material for: Hydrogen Bonding Penalty upon Ligand Binding
Source: PLoS One. 2011 Jun 17;6(6):e19923. doi: 10.1371/journal.pone.0019923 (PMC3117785; doi:10.1371/journal.pone.0019923)
Supplement: Figure S4 — 2D plot of the binding mode of Imatinib. Upon ligand binding, one nitrogen atom of the Imatinib pyrimidine ring (N1) becomes water inaccessible and does not form a new hydrogen bond, leading to a penalty of 1. By contrast, the other nitrogen atom (N2) remains hydrogen bonding with a nearby bound water molecule and thus has no penalty. (DOC) [file pone.0019923.s004.doc]

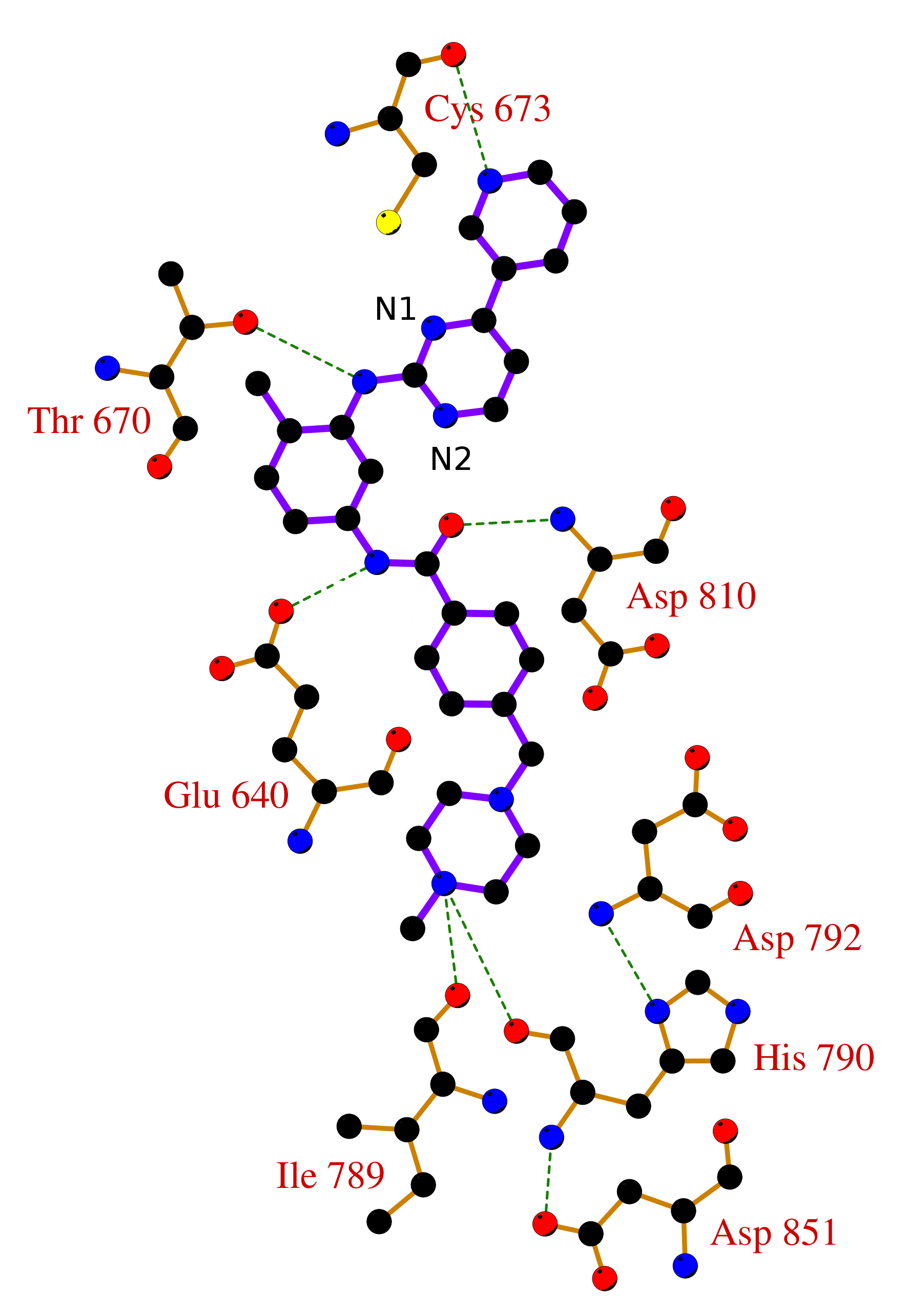


Figure S4: 2D plot of the binding mode of Imatinib. Upon ligand binding, one nitrogen atom of the Imatinib pyrimidine ring (N1) becomes water inaccessible and does not form a new hydrogen bond, leading to a penalty of 1. By contrast, the other nitrogen atom (N2) remains hydrogen bonding with a nearby bound water molecule and thus has no penalty.
