# Supplementary material for: Hydrogen Bonding Penalty upon Ligand Binding
Source: PLoS One. 2011 Jun 17;6(6):e19923. doi: 10.1371/journal.pone.0019923 (PMC3117785; doi:10.1371/journal.pone.0019923)
Supplement: Figure S5 — Poses with the most favorable binding energy of inhibitors of CDK2 (A), EphB4 (B), p38 α (C), Braf (D) and another set of p38α inhibitors (E). The molecules with bonds in red are the binding modes of the corresponding scaffolds in the crystal structures. (DOC) [file pone.0019923.s005.doc]

**
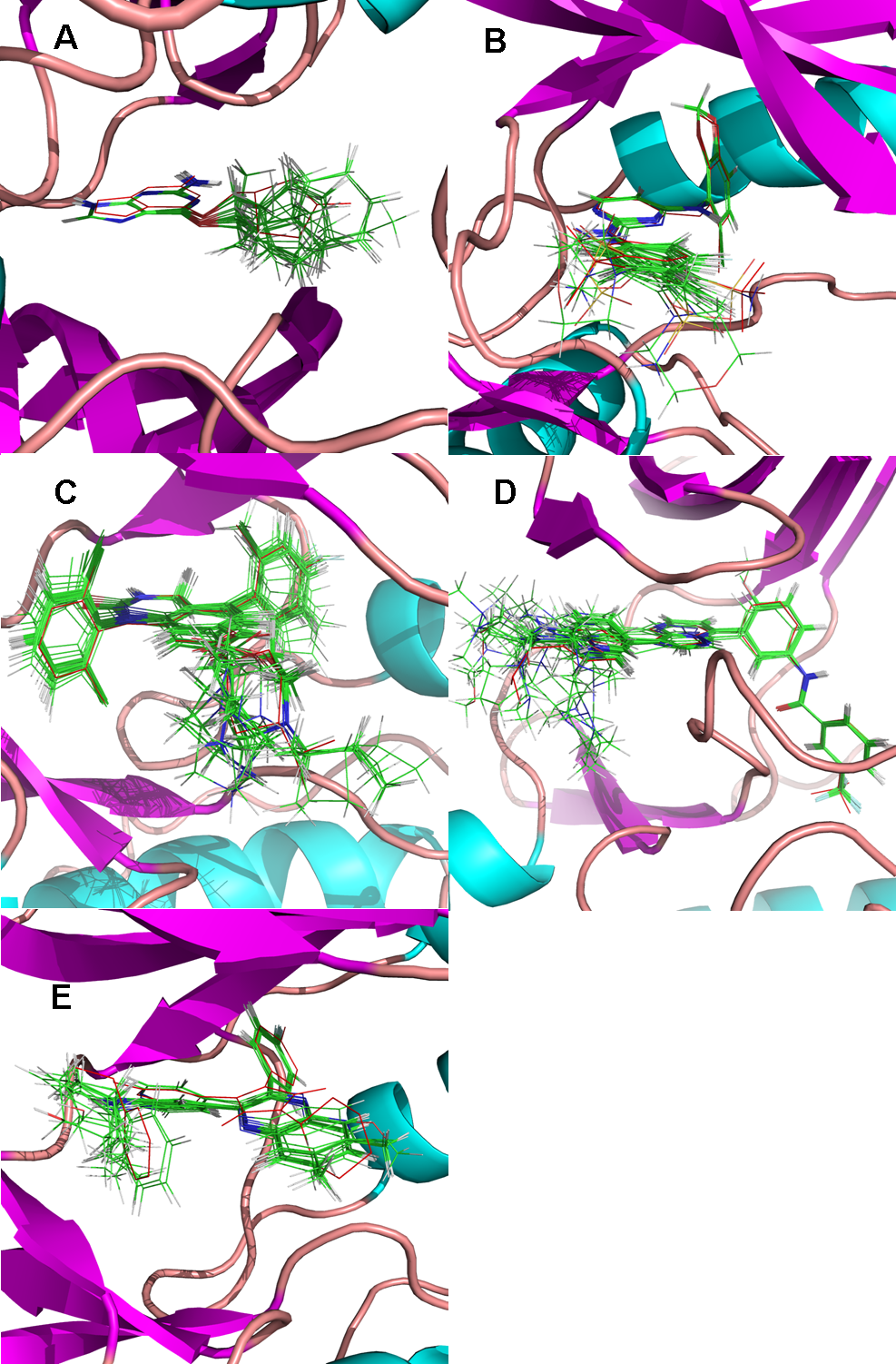
**

**Figure S5.** Poses with the most favorable binding energy of inhibitors of CDK2 (A), EphB4 (B), p38 α (C), Braf (D) and another set of p38α inhibitors (E). The molecules with bonds in red are the binding modes of the corresponding scaffolds in the crystal structures.
