# Supplementary material for: Hydrogen Bonding Penalty upon Ligand Binding
Source: PLoS One. 2011 Jun 17;6(6):e19923. doi: 10.1371/journal.pone.0019923 (PMC3117785; doi:10.1371/journal.pone.0019923)
Supplement: Figure S6 — Distribution of predicted binding affinities by Autodock4 (black) and the proposed scoring function (red) on 74,678 compounds passing the first two filters (HB to Met696 and P HB≤2 kcal/mol). Bin size: 0.1 kcal/mol. (DOC) [file pone.0019923.s006.doc]

**Figure S6.** Distribution of predicted binding affinities by Autodock4 (black) and the proposed scoring function (red) on 74,678 compounds passing the first two filters (HB to Met696 and *P*_HB_ ≤ 2 kcal/mol). Bin size: 0.1 kcal/mol.
