# Supplementary material for: Hydrogen Bonding Penalty upon Ligand Binding
Source: PLoS One. 2011 Jun 17;6(6):e19923. doi: 10.1371/journal.pone.0019923 (PMC3117785; doi:10.1371/journal.pone.0019923)
Supplement: Table S1 — MPEOE partial charge and water solubility of model small molecules used to generate initial guess of hydrogen bonding weights. (DOC) [file pone.0019923.s007.doc]

**Table S1.** MPEOE partial charge and water solubility of model small molecules used to generate initial guess of hydrogen bonding weights

|  | MPEOE partial charge (e) | Solubility in water at 20 ^o^C |
| --- | --- | --- |
| pyridine | N (-0.58) | miscible |
| N-methylacetamide | O (-0.56); H (0.26) | N.A. |
| acetone | O (-0.49) | miscible |
| aniline | H (0.27) | 3.6 g/100ml |
| n-propanol | O (-0.62); H (0.39) | miscible |
| n-butanol | O (-0.62); H (0.39) | 7.7 g/100ml |
| phenol | O (-0.59); H (0.40) | 8.3 g/100ml |
| diethyl ether | O (-0.37) | 6.9 g/100ml |
