# Supplementary material for: Hydrogen Bonding Penalty upon Ligand Binding
Source: PLoS One. 2011 Jun 17;6(6):e19923. doi: 10.1371/journal.pone.0019923 (PMC3117785; doi:10.1371/journal.pone.0019923)
Supplement: Table S2 — Two-parameter LIECE energy and hydrogen bonding penalty on the 24 EphB4 inhibitors. (DOC) [file pone.0019923.s008.doc]

**Table S2.** Two-parameter LIECE energy and hydrogen bonding penalty on the 24 EphB4 inhibitors.

| No. | *P*_HB_ (kcal/mol) | LIECE  (kcal/mol) | Exp. Δ*G*  (kcal/mol) | No. | *P*_HB_  (kcal/mol) | LIECE  (kcal/mol) | Exp. Δ*G*  (kcal/mol) |
| --- | --- | --- | --- | --- | --- | --- | --- |
| **8** | 1.05 | -12.52 | -7.93 | **20** | 1.04 | -12.73 | -7.79 |
| **9** | 1.03 | -12.39 | -7.23 | **21** | 1.09 | -12.06 | -6.40 |
| **10** | 1.07 | -12.74 | -8.10 | **22** | 1.05 | -12.82 | -8.54 |
| **11** | 1.02 | -12.60 | -7.69 | **23** | 1.01 | -12.74 | -8.05 |
| **12** | 1.09 | -12.78 | -6.75 | **24** | 1.49 | -12.72 | -6.07 |
| **13** | 1.04 | -13.08 | -8.02 | **25** | 1.05 | -12.87 | -10.09 |
| **14** | 1.02 | -12.81 | -7.12 | **26** | 1.03 | -12.74 | -8.90 |
| **15** | 2.09 | -12.98 | -6.62 | **27** | 1.43 | -12.95 | -5.99 |
| **16** | 1.04 | -12.99 | -8.65 | **28** | 1.02 | -13.44 | -9.61 |
| **17** | 1.04 | -12.78 | -7.91 | **29** | 1.05 | -13.22 | -8.46 |
| **18** | 1.31 | -12.94 | -7.38 | **31** | 1.03 | -13.98 | -8.51 |
| **19** | 1.03 | -13.01 | -8.45 | **32** | 1.06 | -13.46 | -8.19 |
