# Supplementary material for: Hydrogen Bonding Penalty upon Ligand Binding
Source: PLoS One. 2011 Jun 17;6(6):e19923. doi: 10.1371/journal.pone.0019923 (PMC3117785; doi:10.1371/journal.pone.0019923)
Supplement: File S1 — Probing hydrogen bonds formed with implicit water. (DOC) [file pone.0019923.s009.doc]

**Scheme 1.** Schematic view of probing hydrogen bonds formed with implicit water. Three shells around each donor or acceptor were generated with the radius being *r*_sol_-0.15, *r*_sol_, and *r*_sol_+0.15 Å, respectively. In the current study, 2200 dots were distributed over the surface of each shell, with each dot representing the probability of a hydrogen bond with implicit water. Dots with the angle Θ less than 90^o^ or within the inner shell of other atoms will be removed. Any dot left indicates hydrogen bonding with water remains.

**Probing hydrogen bonds formed with bound water molecules**

Among the interfacial water molecules observed in the HIV-protease-ligand complexes, Water 301 has been found to bridge the gaps between flaps of HIV-1 protease and inhibitors. This water molecule forms two hydrogen bonds with NH groups of Ile50 and Ile150 as an acceptor, meanwhile forms two additional hydrogen bonds with the carbonyl groups of the inhibitor as a donor (Figure 1). Water 301 is observed in nearly all HIV-1 protease-ligand complexes, except in the case it is displaced deliberately [1].

To see whether the parameters employed can rigorously probe hydrogen bonds formed with implicit bound water molecules, four HIV-1 protease crystal structures were tested. As illustrated in Figure 1, each of the four polar atoms forming hydrogen bonds with Water 301 is calculated as water accessible, and the calculated water molecules meet at the position of Water 301, indicative of one water molecule bridging all the four polar atoms. The number of predicted dots as potential water positions also represents the entropy of the water molecule. Compared with the other two crystal water molecules, the number of predicted dots for Water 301 is fewer, consistent with the fact that Water 301 is highly constrained. Hydrogen bonding penalty for each complex as well as partial contribution from the protein and ligand was given in Table 1. Only one of the catalytic aspartyl diad (Asp25 and Asp125) was deprotonated, as evidenced by NMR study [2].


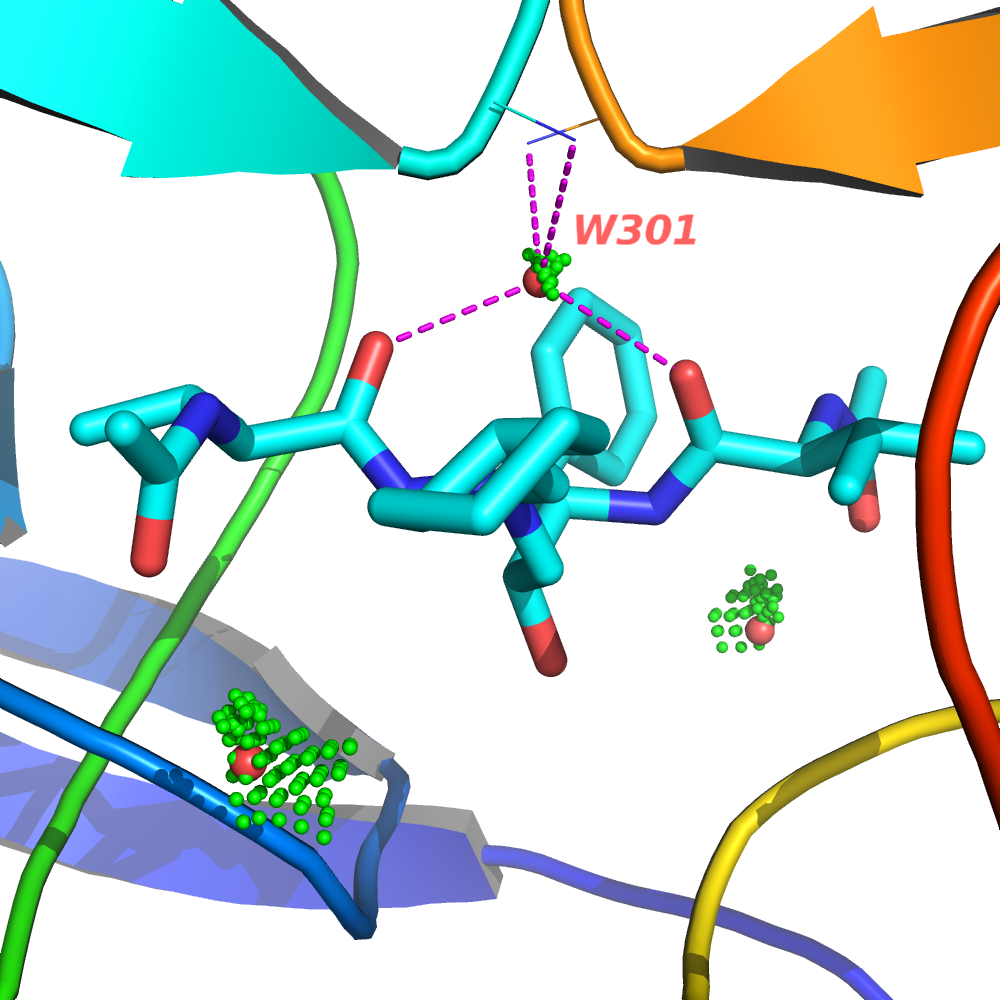


**Figure 1.** Cartoon representation of HIV-1 protease ligand site (1HIH) with crystal (red dots) and calculated (green) water oxygen atoms.

**Table 1.** Hydrogen bonding penalty of HIV-1 protease complexes.

| PDB code | Resolution (Å) | *P*_HB_ | | |
| --- | --- | --- | --- | --- |
|  |  | Protein | Ligand | Total |
| 1HXW | 1.80 | 0.70 | 0.68 | 1.38 |
| 1HXB | 2.30 | 0.58 | 0.66 | 1.23 |
| 1HPX | 2.00 | 1.00 | 0.56 | 1.56 |
| 1HIH | 2.20 | 1.04 | 0.11 | 1.16 |

**References**

1. Lam PY, Jadhav PK, Eyermann CJ, Hodge CN, Ru Y, et al. (1994) Rational design of potent, bioavailable, nonpeptide cyclic ureas as HIV protease inhibitors. Science 263: 380-384.

2. Wang YX, Freedberg DI, Yamazaki T, Wingfield PT, Stahl SJ, et al. (1996) Solution NMR evidence that the HIV-1 protease catalytic aspartyl groups have different ionization states in the complex formed with the asymmetric drug KNI-272. Biochemistry 35: 9945-9950.
