# Supplementary material for: Hydrogen Bonding Penalty upon Ligand Binding
Source: PLoS One. 2011 Jun 17;6(6):e19923. doi: 10.1371/journal.pone.0019923 (PMC3117785; doi:10.1371/journal.pone.0019923)
Supplement: File S2 — Hydrogen bonding penalty of the 100 kinase complex structures. (DOC) [file pone.0019923.s010.doc]

**Figure 1.** Number of donors/acceptors against molecular weight of inhibitors complexed in the 100 kinase crystal structures. Colored according to hydrogen bonding penalty.

**Figure 2.** Statistics of hydrogen bonding penalty on the 100 kinase complexes.

**Table 1. Hydrogen bonding penalty of the 100 kinase complex structures.**

| Protein | PDB code | *P*_HB_ | Protein | PDB code | *P*_HB_ | Protein | PDB code | *P*_HB_ |
| --- | --- | --- | --- | --- | --- | --- | --- | --- |
| CDK2 | 1gz8 | 0.38 | Abl | 1iep | 1.23 | p38α | 1ove | 1.02 |
|  | 1h0v | 0.71 |  | 3kfa | 1.57 |  | 1oz1 | 0.00 |
|  | 1h0w | 0.47 |  | 1opj | 1.12 |  | 1w82 | 1.16 |
|  | 3lfn | 1.04 |  | 2f4j | 1.83 |  | 1w84 | 0.21 |
|  | 3lfq | 0.24 |  | 2g2h | 1.50 |  | 1yqj | 0.00 |
|  | 3lfs | 0.71 |  | 2gqg | 0.51 |  | 1ywr | 1.11 |
|  | 3ig7 | 0.06 |  | 3dk3 | 0.52 |  | 1zzl | 1.27 |
|  | 3igg | 0.26 |  | 3dk6 | 0.52 |  | 3gc7 | 0.02 |
|  | 1aq1 | 0.45 |  | 3cs9 | 0.22 |  | 3gcq | 0.90 |
|  | 1e1v | 0.74 |  | 2qoh | 0.61 |  | 3flq | 1.01 |
|  | 1e1x | 0.71 |  | 2v7a | 0.48 |  | 3fls | 1.00 |
|  | 1jvp | 1.16 |  | 2z60 | 0.77 |  | 3flw | 1.01 |
|  | 1h1r | 1.57 |  | 2e2b | 1.47 | AurA | 3d15 | 1.11 |
|  | 2r3f | 0.49 |  | 2hyy | 1.34 |  | 2c6e | 1.76 |
|  | 2r3h | 0.11 |  | 2hz0 | 0.73 |  | 3d2k | 1.12 |
|  | 3le6 | 0.05 |  | 2hzi | 1.50 |  | 3dj6 | 0.80 |
|  | 1ke5 | 0.70 | Lck | 3bym | 2.06 |  | 3e5a | 1.00 |
| EphB4 | 2vwu | 1.51 |  | 3bys | 0.21 |  | 3d14 | 1.20 |
|  | 2vwx | 1.18 |  | 2of2 | 0.83 |  | 3daj | 0.00 |
|  | 2vwy | 1.96 |  | 2ofu | 1.90 | EGFR | 3bel | 1.00 |
|  | 2vwz | 0.95 |  | 2ofv | 0.54 |  | 1xkk | 0.20 |
|  | 2vx0 | 0.90 |  | 2og8 | 0.74 |  | 2rgp | 1.58 |
|  | 2vx1 | 0.98 |  | 1qpj | 0.99 | Tie2 | 2oo8 | 0.53 |
|  | 2vwv | 1.18 | JAK2 | 3e62 | 0.13 |  | 2p4i | 0.96 |
|  | 2vww | 0.96 |  | 3e63 | 0.02 | PAK4 | 2x4z | 1.02 |
| JNK3 | 1pmn | 1.92 |  | 3e64 | 0.52 |  | 2cdz | 0.10 |
|  | 1pmv | 0.30 |  | 3iok | 1.19 | Kit | 1t46 | 1.70 |
|  | 2b1p | 0.16 |  | 3kck | 0.56 |  | 2oiq | 0.12 |
|  | 2ok1 | 0.27 |  | 3jy9 | 0.29 | BTK | 3gen | 0.81 |
|  | 2p33 | 1.29 |  | 3lpb | 2.01 |  | 3k54 | 1.02 |
|  | 2r9s | 0.32 | Ret | 2x2l | 0.00 | Hck | 1qcf | 0.61 |
|  | 3g90 | 2.04 |  | 2ivu | 1.08 |  | 2hk5 | 0.28 |
|  | 3fi3 | 0.39 |  | 2ivv | 0.41 |  | | |
|  | 3g9l | 1.46 |  | 2x2m | 0.11 |  | | |
