# Supplementary material for: Hydrogen Bonding Penalty upon Ligand Binding
Source: PLoS One. 2011 Jun 17;6(6):e19923. doi: 10.1371/journal.pone.0019923 (PMC3117785; doi:10.1371/journal.pone.0019923)
Supplement: File S3 — Ranking the strength of individual hydrogen bonds in DNA base pairs. (DOC) [file pone.0019923.s011.doc]

**Ranking the strength of individual hydrogen bonds in DNA base pairs**

The three hydrogen bonds **a**, **b** and **c** in DNA bases pairs (Scheme 1) were ranked according to the hydrogen bonding strength calculated by *f*_hb_*(*w*_A_ + *w*_D_). Different approaches [1,2,3,4,5,6] showed substantial discrepancy (Table 1). In the case of G-C complex, larger discrepancy was observed, presumably because the energies of the three hydrogen bonds are very close. However, in the case of A-T complex, five methods including us gave the same ordering, with **b** being the strongest, and all methods predicted **c** as the weakest.

**Scheme 1.** H-bonded complexes of nucleic acid pairs

**Table 1. Ranking of the individual H-bonds strengths in the G-C and A-T base pairs (Scheme 1) on the basis of literature data and our results.**

| Method of characterizing the H-bonds | Calculation level | G-C | A-T |
| --- | --- | --- | --- |
| Rotation [1] | B3LYP/D95** | **c**>**b**>**a** | **a**>**b**>**c** |
| Compliance constants [2] | B3LYP/6-311++G** | **b**>**a**>**c** | **b**>**a**>**c** |
| Atom replacement [3] | B3LYP/6-311++G** | **a**>**c**>**b** | **b**>**a**>**c** |
| EML equation [4] for experimental geometry | B3LYP/6-311++G** | **a**>**b**>**c** | **a**>**b**>**c** |
| E_H…B_ vs. ρ_CP_ relation [5] | B3LYP/6-311++G** | **a**>**c**>**b** | **b**>**a**>**c** |
| NBO [6] | B3P86/6-311++G** | **a**>**b**>**c** | **b**>**a**>**c** |
| Hydrogen bonding weights | N.A. | **b**>**a**=**c** | **b**>**a**>**c** |

**References**

1. Asensio A, Kobko N, Dannenberg JJ (2003) Cooperative hydrogen-bonding in adenine-thymine and guanine-cytosine base pairs. Density functional theory and Moller-Plesset molecular orbital study. Journal of Physical Chemistry A 107: 6441-6443.

2. Grunenberg J (2004) Direct assessment of interresidue forces in Watson-Crick base pairs using theoretical compliance constants. J Am Chem Soc 126: 16310-16311.

3. Dong H, Hua W, Li S (2007) Estimation on the individual hydrogen-bond strength in molecules with multiple hydrogen bonds. J Phys Chem A 111: 2941-2945.

4. Matta CF, Castillo N, Boyd RJ (2006) Extended weak bonding interactions in DNA: pi-stacking (base-base), base-backbone, and backbone-backbone interactions. J Phys Chem B 110: 563-578.

5. Ebrahimi A, Khorassani SMH, Delarami H (2009) Estimation of individual binding energies in some dimers involving multiple hydrogen bonds using topological properties of electron charge density. Chemical Physics 365: 18-23.

6. Szatylowicz H, Sadlej-Sosnowska N (2010) Characterizing the Strength of Individual Hydrogen Bonds in DNA Base Pairs. J Chem Inf Model.
